# First Association of Interleukin 12 Receptor Beta 1 Deficiency with Sjögren’s Syndrome

**DOI:** 10.3389/fimmu.2017.00885

**Published:** 2017-07-28

**Authors:** Georgios Sogkas, Faranaz Atschekzei, Vivien Schacht, Christian von Falck, Alexandra Jablonka, Roland Jacobs, Matthias Stoll, Torsten Witte, Reinhold E. Schmidt

**Affiliations:** ^1^Division of Immunology and Rheumatology, Hannover Medical University, Hanover, Germany; ^2^Division of Dermatology, Hannover Medical University, Hanover, Germany; ^3^Institute for Diagnostic and Interventional Radiology, Hannover Medical University, Hanover, Germany

**Keywords:** interleukin-12, interleukin-12 receptor beta 1 subunit, Sjögren’s syndrome, primary immunodeficiency, autoimmunity, Mendelian susceptibility to mycobacterial disease, interleukin-12 receptor beta 1 subunit deficiency

## Abstract

**Introduction:**

Interleukin 12 receptor beta 1 (IL12Rβ1) deficiency is a primary immunodeficiency resulting mainly in susceptibility to opportunistic infection by non-tuberculous, environmental mycobacteria and severe infection caused by *Salmonella* spp. Till now, less than 300 patients with IL12Rβ1 deficiency have been reported. Among them, only three have been described to develop autoimmunity.

**Case presentation:**

We present the case of a 50-year-old male with IL12Rβ1 deficiency due to compound *heterozygosity [c. 1623_1624delGCinsTT (pGln542Stop) and c.1791* + *2T* > *C (donor splice site)]*, who—18 months after diagnosis of disseminated BCGitis—presented with recurrent fever and sicca syndrome. No indication of an infectious origin of these symptoms could be found at that point. The diagnosis of a Sjögren’s syndrome (SS) was made on the basis of fulfilled American-European consensus classification criteria, including a positive minor salivary gland biopsy.

**Conclusion:**

Apart from persistent antigenic stimulation, which may drive autoimmune inflammation in primary immunodeficiency, evidence on the involvement of interleukin 12 in pathogenesis of SS suggests that the same immunological mechanism may underlie both defense against infection and the maintenance of tolerance. To our knowledge, this is the first report of a case of autoimmunity in the form of SS in a patient with a primary immunodeficiency and one of the rare cases of IL12Rβ1 deficiency with manifested autoimmunity.

## Introduction

The capability to produce or respond to interferon γ (IFNγ) is important in controlling infection by intracellular bacteria such as mycobacteria and *Salmonella* spp. (1, 2). Mutations in genes encoding for type I cytokines and molecules involved in their signaling have been identified in patients with infection due to environmental mycobacteria. In particular, mutations of the *IL12RB1*-gene resulting in Interleukin 12 receptor beta 1 (IL12Rβ1) deficiency are the most common cause of Mendelian susceptibility to mycobacterial disease (MSMD) (2). IL12Rβ1 deficiency follows an autosomal recessive pattern of inheritance, although the clinical outcome of IL12Rβ1-deficient individuals, carrying two mutant alleles, is variable, ranging from early death to an asymptomatic outcome. This incomplete penetrance may reflect differences in the genetic background of IL12Rβ1-deficient individuals and/or the environmental exposure to relevant pathogens. According to the study by de Beaucoudrey et al., including over 140 IL12Rβ1-deficient individuals, in more than 90% of cases, IL12Rβ1-deficiency was symptomatic with mycobacterial infections representing the most common phenotype, found in 77% of IL12Rβ1-deficient individuals. The second most common infection was Salmonellosis, affecting 40% of IL12Rβ1-deficient individuals. Disease onset occurred in early and middle childhood in most cases and a lethal outcome was observed in up to a third of patients (3).

Interleukin 12 receptor beta 1 physically associates with p40, which is a common subunit of interleukin 12 (IL-12) and interleukin 23 (IL-23) (4). IL-12 and IL-23 are proinflammatory cytokines, whose biological activities depend on IL12Rβ1. IL12Rβ1 is bound to the non-receptor protein tyrosine kinase 2, whereas IL12Rβ2—its IL-12 receptor (IL-12R) counterpart chain—is bound to the Janus kinase 2. Activation of IL-12R results in the phosphorylation, homodimerization, and nuclear translocation of the signal transducer and activator of transcription 4 (STAT4), which induces the expression of IFNγ. IL12Rβ1 deficiency and the consequent reduced IL-12 responsivity result in impaired production of IFNγ by NK cells and T cells, which impairs control of infection by mycobacteria and other intracellular bacteria (1).

Primary immunodeficiency diseases are often associated with autoimmunity, which may reflect the fact that common mechanisms account for both defense against possible pathogens and the maintenance of tolerance (5, 6). Apart from that, it has been suggested that persistent or recurrent antigenic stimulation as a consequence of infectious diseases in patients with primary immunodeficiency may cause or contribute to breaking of tolerance.

In contrast to other primary immunodeficiency diseases, there is only scarce evidence for association of defects of the IL*-*12*/*IFN*-*γ axis, including IL12Rβ1 deficiency with autoimmunity. Here, we present the case of a 50-year-old male with IL12Rβ1-deficiency, who presented with fever episodes and sicca syndrome 18 months after starting of an antimycobacterial therapy due to the diagnosis of a disseminated infection with Bacillus Calmette–Guérin (BCG). In the absence of evidence of infectious origin of these symptoms and on the basis of fulfilled American-European consensus classification criteria (7), we diagnosed a Sjögren’s syndrome (SS).

## Case Report

A male infant, only child born to non-consanguineous Caucasian parents of German descent in 1966, developed after birth an inguinal lymphadenopathy, which was treated with a single antibiotic for approximately 2 weeks. At the age of 20 years, he developed a left-sided axillary lymphadenopathy, which led to the dissection of an axillary lymph node. Histological examination yielded no relevant pathological findings. A 2-week-treatment with antibiotics resulted in regression of the lymphadenopathy.

**Figure 1:**
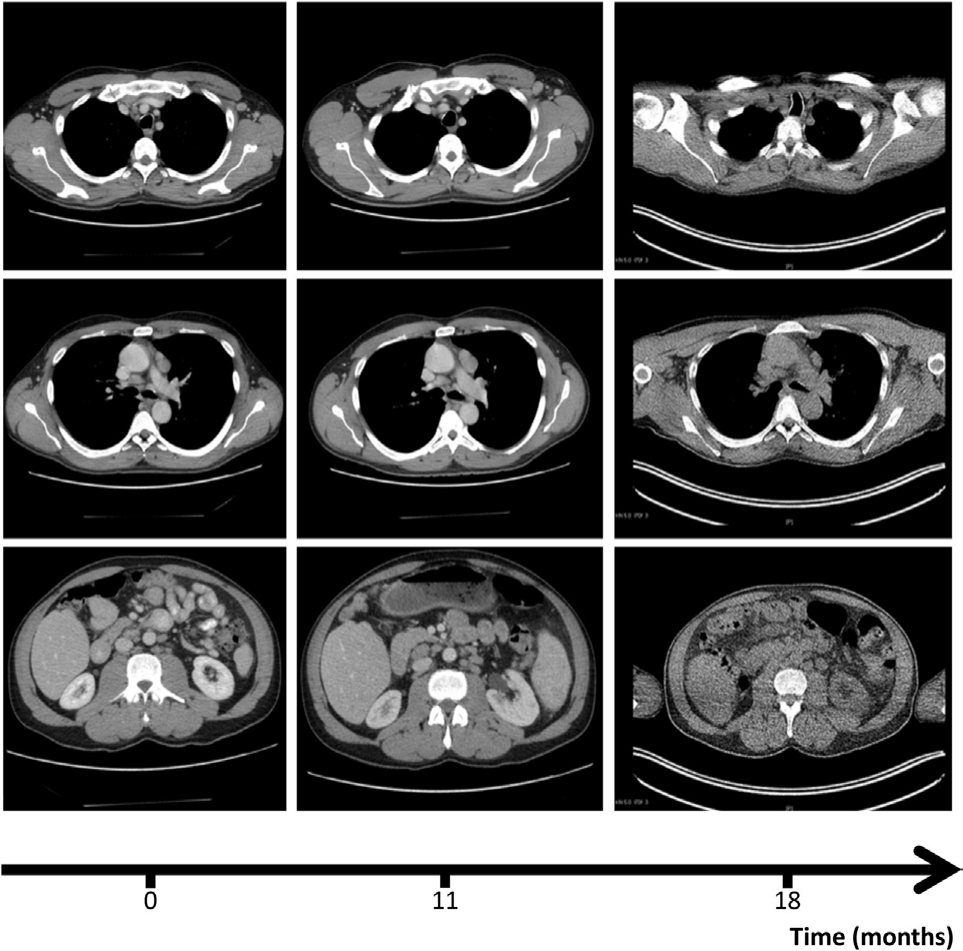
Computer tomography (CT) scans, revealing axillary (upper row), mediastinal (mid row), and abdominal lympadenopathy (lower row) at the three different time points: diagnosis of disseminated infection with Bacillus Calmette–Guérin (0, left column), escalation of antimycobacterial therapy (11, mid column), and diagnosis of Sjögren’s syndrome (18, right column). CT scan of right row was performed in the context of a positron emission tomography/CT (see Figure 4).

**Figure 2:**
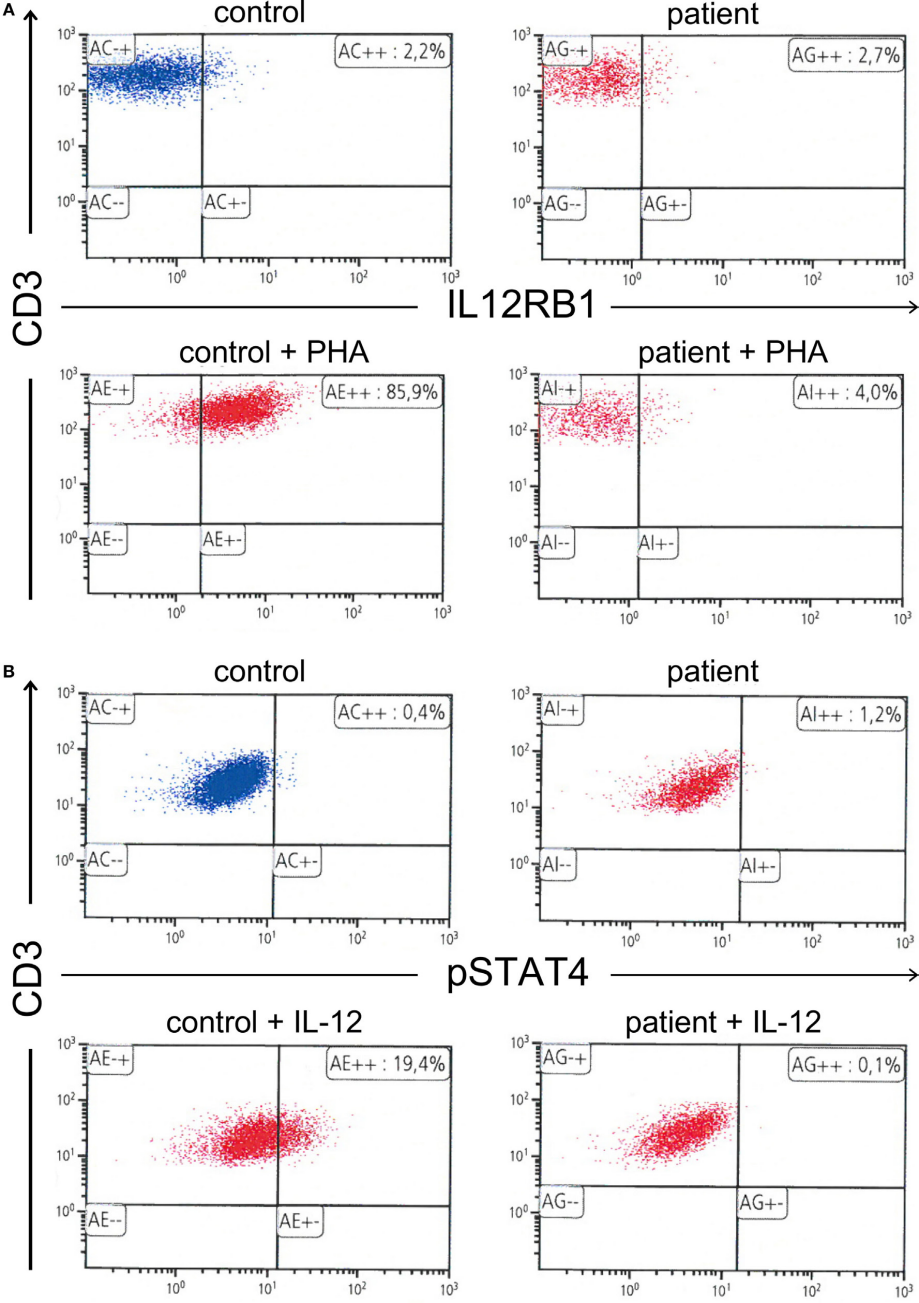
**(A)** Absence of interleukin 12 receptor beta 1 expression on the surface of phytohemagglutinin-stimulated T cells. **(B)** Further, absence of signal transducer and activator of transcription 4 phosphorylation in interleukin 12 (IL-12)-stimulated T cells.

At the age of 47, the patient developed a disseminated lymphadenopathy; a computer tomography (CT) scan revealed a pathological enlargement of abdominal, mediastinal, supraclavicular, and axillary lymph nodes (Figure 1, left column). An infection by BCG was diagnosed after a biopsy of a right axillary lymph node in a peripheral hospital of our region. Detection of BCG was achieved by culture of the biopsy sample and analysis of the genomic “region of difference 1” (RD1) of the cultured mycobacteria. In line with this diagnosis, is the fact that the patient as an infant had received vaccination with BCG. An antimycobacterial therapy with rifampicin, isoniazid, ethambutol, and azithromycin was started. A macrolide antibiotic, i.e., azithromycin was added to achieve coverage of other environmental mycobacteria. At that time, the patient was referred to our department for Clinical Immunology due to a suspected immunodeficiency. On admission, the C-reactive protein (CRP) was elevated (82 mg/l, normal <5 mg/l). The rest of routine laboratory tests were normal, except for a slight increase of gammaglutamyltransferase (gGT, 100 U/l, normal <55 U/l). A serum protein electrophoresis revealed a polyclonal hypergammaglobulinemia. Immunoglobulin classes revealed increased levels for serum immunoglobulin G (27.7 g/l, normal range: 7–16 g/l). Serum and urine immunofixation were negative. Immunophenotyping of blood lymphocyte subsets revealed a slight absolute reduction in B cell count with normal absolute and relative counts for the rest of lymphocyte subsets and an increased expression of human leukocyte antigen-antigen D related (HLA-DR), indicating lymphocyte activation. T cell activation with distinct stimuli (including interleukin 2, anti-CD3-antibody, phytohemagglutinin (PHA), concanavalin A, and pokeweed mitogen) resulted in an adequate T cell proliferation. Testing of granulocytes for oxidative burst and phagocytic potential yielded adequate responses. Searching for the underlying cause of a BCGitis, we also tested for an abnormality of the IL*-*12*/*IFNγ-pathway, which showed the absence of IL12Rβ1 on the surface of PHA-stimulated T cells. Further, there was no STAT4 phosphorylation after T cell stimulation with IL-12. Results of flow cytometric analysis of IL12Rβ1 expression/STAT4 phosphorylation and the rest of immunological investigations are presented in Figure 2 and Table 1, respectively. We then performed genetic analysis focused on the *IL12RB1*-gene (NM_005535), which revealed a compound heterozygosity [*c. 1623_1624delGCinsTT* (*pGln542Stop*) and *c.1791* + *2T* > *C* (donor splice site)], explaining the absence of IL12Rβ1 expression (2).

**Table 1:** Immunological findings on admission, directly after diagnosis of disseminated infection with Bacillus Calmette–Guérin in a 47-year-old male with interleukin 12 receptor beta 1 deficiency.

Immunological test	Value	Normal range (or control value)
**Serum Ig concentration**		
IgG (g/l)	27.7	7.0–16.0
IgA (g/l)	4.4	0.7–4.0
IgM (g/l)	1.8	0.4–2.3
IgE (IE/ml)	680	1–100
**Complement**		
Total complement activity CH50 (%)	>165	90–150
C3c (g/l)	1.91	0.9–1.8
C4 (g/l)	0.14	0.1–0.4
**Lymphocyte subset analysis**		
Lymphocyte count (cells/μl)	4,030	1,000–2,800/μl
CD3+ (relative %/cells/μl)	89%/3,909	700–2,100
CD3+/CD4+ (relative %/cells/μl)	34%/1,370	500–1,400
CD3+/CD8+ (relative %/cells/μl)	44%/1,773	200–900
CD3+/human leukocyte antigen-antigen D related+ (relative %/cells/μl)	13%/524	30–200
CD3−/CD16+/CD56+ (relative %/cells/μl)	8%/322	90–600
CD19+ (relative %/cells/μl)	2%/81	100–500
**Lymphocyte proliferation assay**		
Medium control (cells/μl)	2,741	2,379
Phytohemagglutinin (cells/μl)	42,100	34,295
Concanavalin A (cells/μl)	26,008	7,928
Pokeweed mitogen (cells/μl)	13,487	10,151
Tuberculin purified protein derivative (cells/μl)	2,580	1,484
Interleukin 2 (cells/μl)	4,887	2,505
Isotype control (cells/μl)	601	218
Anti-CD3-antibody (cells/μl)	26,326	24,235
**Granulocyte phenotyping**		
CD16 (%)	98	99
CD32 (%)	99	99
CD64 (%)	3	1
CD18 (%)	100	100
CD11b (%)	100	100
CD15s (%)	100	99
**Granulocyte function tests**		
Phagocytosis of *E. coli* (%)	94	96
Oxidative burst induction (oxidation of 2′,7′*-*dichlorofluorescin diacetate)		
Medium control (%)	2	1
*N*-formylmethionyl-leucyl-phenylalanine (%)	4	3
*E. coli* (%)	100	98
Phorbol 12-myristate 13-acetate (%)	100	100

Two months later, ethambutol was stopped and 8 months later, B symptoms (fever, weight loss, and night sweats), which were present at the first presentation of the patient in our clinic, reappeared. CRP, which had been steadily decreasing after beginning of the antimycobacterial therapy, was again elevated (78 mg/l). A CT-scan revealed persistence of the lymphadenopathy (Figure 1, mid column), so that we assumed a relapse of the BCGitis. We, therefore, escalated the antimycobacterial therapy by adding ethambutol, terizidone, and moxifloxacin to the at that time point ongoing therapy with rifampicin, isoniazid, and azithromycin.

**Figure 3:**
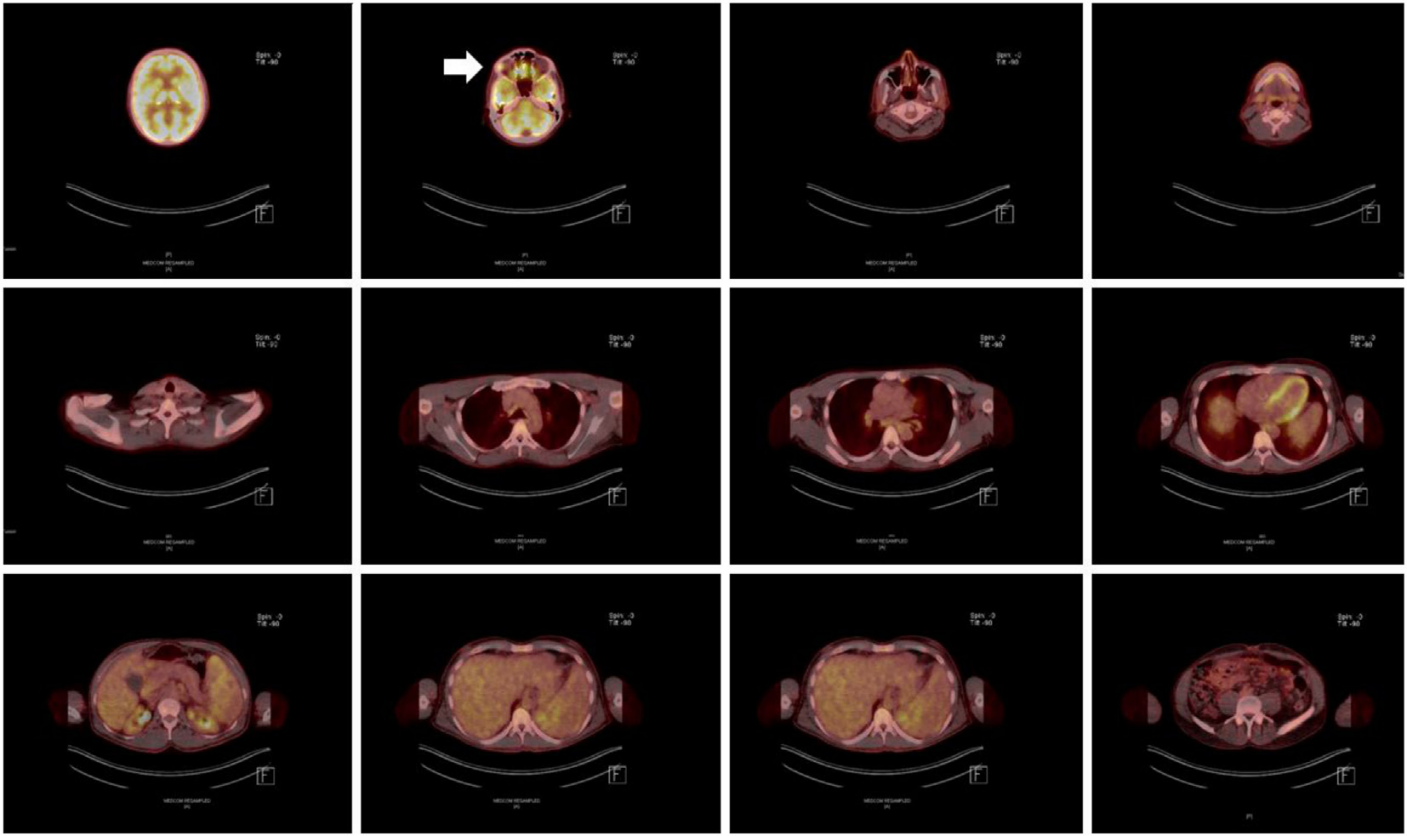
Positron emission tomography/computer tomography scan after completion of an 18-month antimycobacterial therapy, including a 6-month 6-drug regimen, revealing significant homogenous hypermetabolism in the area of the right lacrimal gland (marked with an arrow).

**Figure 4:**
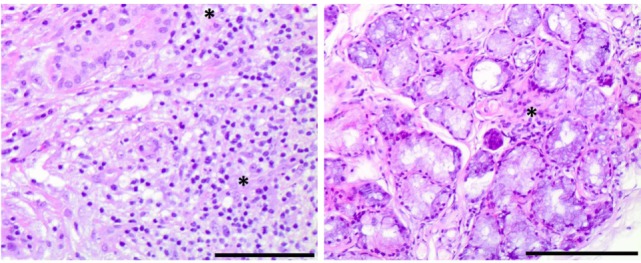
Histological findings of Sjögren’s syndrome in an interleukin 12 receptor beta 1-deficient male. Right panel: lymphocytic infiltrates with periductal distribution in a lacrimal gland biopsy section, stained with hematoxylin and eosin (10× magnification, bar size: 0.5 µg) Left panel: focal lymphocytic infiltration in a minor salivary gland biopsy section, stained with hematoxylin and eosin (10× magnification, bar size: 0.5 µg).

Despite initial regression of B symptoms under the six-drug antimycobacterial therapy, 6 months later, the patient developed once more fever with night sweating. Serologic inflammation activity was again increased as exemplified by elevated CRP (117 mg/l). In a follow-up, we performed a CT scan combined with positron emission tomography. We detected a regression of the lymphadenopathy (Figure 1, right column), with diffuse increased uptake of F-18 fluoro-2-deoxyglucose (18FDG) in bone marrow (Figure 3). Increased uptake of 18FDG was also localized in the area of the right lacrimal gland. With these findings, we performed a bone marrow biopsy, which revealed a minor hyperplastic granulopoiesis and megakaryopoiesis with a non-specific inflammatory reaction. Further, a biopsy of the right lacrimal gland revealed focal lymphocytic infiltrates with periductal distribution (Figure 4). Microbiological testing of bone marrow and lacrimal gland samples, including polymerase chain reactions to detect mycobacteria, remained negative.

Taken the absence of evidence for an infectious cause of the aforementioned symptoms and findings, we suspected an autoimmune inflammation. Serum immunological tests showed an increased titer of antinuclear antibodies (ANA 1:320, normal <1:160) with no specification in the extractable nuclear antigen screening test. The test for rheumatoid factor was highly positive (2,750 IE/ml, normal <15.9 IE/ml) with a negative test for anti-cyclic citrullinated peptide antibody. Further, anti-alpha-fodrin antibodies of IgG and IgA class could be detected (IgA anti-alpha-fodrin 16 U/ml, normal <15 U/ml, and IgG anti-alpha-fodrin 38, normal <15 U/ml). The rest of performed autoantibody tests [anti-double-stranded DNA, antineutrophil cytoplasmic antibodies (pANCA and cANCA), IgG and IgM anti-cardiolipin, anti-Smith antigen (Sm), anti-ribonucleoprotein, anti-Ro (SSA), anti-La (SSB), anti-Jo1, and anti-topoisomerase I (Scl-70)] were negative. The patient complained of xerophthalmia, which could be confirmed by a positive Schirmer’s test. Suspecting a SS, we also performed a labial salivary gland biopsy, which revealed a focal lymphocytic sialadenitis of grade 3 according to the Chisholm–Mason classification (Figure 4). On the basis of fulfilled American-European consensus classification criteria (chronic xerophthalmia, positive Schirmer’s test, positive rheumatoid factor and ANA titer of 1:320, positive labial salivary gland biopsy), we made the diagnosis of a SS and started a treatment with methotrexate (p.o. 15 mg 1×/week) and prednisolone (initially 20 mg and after a week gradual dose reduction till a maintenance dose of 5 mg daily). The intensified 6-drug antimycobacterial regimen was discontinued. Starting an immunosuppressive therapy in the presence of a primary immunodeficiency was done with caution, under close monitoring of the patient in the hospital setting. Considering the absence of evidence of a residual infection with BCG after completion of an 18-month antimycobacterial regimen, the diagnosis of SS and the sustained response to the immusuppressive therapy with maintenance of normal body temperature and CRP reduction as well as the rarity of recurrence or of multiple mycobacterial infections in case of IL12Rβ1-deficiency, especially after an infection with BCG (3), we did not start a prophylactic antimycobacterial therapy. However, we suggested close monitoring though family physician, including testing for inflammatory markers. In the follow-up in our outpatient clinic, 2 and 8 months after starting the treatment with methotrexate the patient remained afebrile and the CRP was reduced (46 mg/l).

## Discussion

We describe a patient with an inherited IL12Rβ1 deficiency, who developed SS approximately 18 months after the diagnosis of a disseminated infection with BCG. A few hundred patients with IL12Rβ1 deficiency have been reported in the literature (3). To our knowledge, till now only three patients with IL12Rβ1-deficiency and clinically significant autoimmune manifestations, including SLE-like disease and autoimmune hemolytic anemia, have been presented (8, 9). This is the first association of a primary immunodeficiency with autoimmunity in the form of SS and a very rare case of manifested autoimmunity in case of IL12Rβ1-deficiency.

Human and mouse-derived evidence implicates IL-12-signaling in the development of SS-like/SS-autoimmunity. *Il-12rb2*-knockout mice, which produce but cannot respond to IL-12, develop SS-like disease with lymphoid infiltration involving the salivary glands and ANA-positivity in serum (10). Interestingly, autoimmunity develops late in these mice, starting at the age of 4 months and worsens as the mice get older. Although not a direct model of IL12Rβ1-deficiency, as IL12Rβ2 is an exclusive constituent chain of IL-12 receptor and not of IL-23 receptor, *il-12rb2*-knockout mice apart from developing late–onset autoimmunity, exhibit similarly to human IL12Rβ1-deficiency a propensity to infection with intracellular pathogens (11). The largest available genome-wide association study on SS recognized variants in genomic regions encoding for constituents of the type I cytokine pathway, such as *IL12A* (encoding the p35 subunits of IL12), *IRF5* (encoding a transcription factor that activates among others the gene for the p40 subunit of IL-12), and *STAT4* (encoding the homonymous transcription factor that activates IFNγ expression) as risk factors in SS (12). Further, autoantibodies against IL-12, which exhibited a neutralizing effect, were found in a subset of patients with primary SS (13). In contrast to the aforementioned findings, suggesting a regulatory role for IL-12 signaling in SS, mice overexpressing IL-12, have been shown to develop SS-like disease, including lymphocytic infiltration of lacrimal glands and anti-SSB/La antibody positivity (14). This may reflect the fact that SS is a heterogeneous disease, with different—even diverging—pathways involved in its pathogenesis.

Further, the present case exemplifies the already described association between mycobacterial infections and autoimmune phenomena, including the emergence of diverse autoantibodies, such as ANA, antineutrophil cytoplasmic antibodies, and anti-SSA/Ro antibodies (15). Recently, Chao et al. have described a significant association between non-tuberculous mycobacterial infection and SS (16), which can be reflecting the pathogenic involvement of mycobacterial infections in SS. However, the fact that non-tuberculous mycobacterial infections, but not infection with mycobacterium tuberculosis associated with increased risk for SS makes it is tempting to speculate that some of these patients may have had an abnormality in the IL*-*12*/*IFNγ-pathway, and especially in IL-12 signaling.

Taken the existing literature, the association between IL12Rβ1 deficiency and SS can be reflecting the regulatory role of IL-12. However, before we can be sure, more cases of adult patients with IL12Rβ1-deficiency need to be investigated. Apart from evidence on the pathogenic involvement of IL-12 in SS, persistent antigenic stimulation as a consequence of recurrent or persistent infection—especially of a mycobacterial infection—in IL12Rβ1-deficiency, may contribute or lead to autoimmunity (6).

## Concluding Remarks

This is the first report of a case of autoimmunity in form of SS in a patient with a primary immunodeficiency and a rare case of manifested autoimmunity in a patient with IL12Rβ1 deficiency, suggesting that the same immunological mechanisms may underlie both defense against infection and the maintenance of tolerance.

## Ethics Statement

This is a report on a single patient, which complies with the Declaration of Helsinki. This study was carried out in accordance with the recommendations of the Ethic committee of the Hannover Medical University. The patient gave a written informed consent to publish the report.

## Author Contributions

GS wrote the paper under the guidance of TW and RS. RS, TW, MS, GS, VS, and AJ were involved in the diagnostic and/or therapeutic course. RJ and AF were performed the laboratory studies for the diagnosis of IL12RB12 deficiency. All coauthors revised the paper and approved its final version for publication.

## Conflict of Interest Statement

The authors declare that the research was conducted in the absence of any commercial or financial relationships that could be construed as a potential conflict of interest.
